# Comparative analysis of miRNA profile in human dendritic cells infected with respiratory syncytial virus and human metapneumovirus

**DOI:** 10.1186/s13104-018-3541-0

**Published:** 2018-07-03

**Authors:** Ma. Del Rocio Baños-Lara, Jovanny Zabaleta, Jone Garai, Melody Baddoo, Antonieta Guerrero-Plata

**Affiliations:** 1 Department of Pathobiological Sciences, Louisiana State University, Baton Rouge, LA 70803 USA; 2 Department of Pediatrics, Louisiana State University Health Sciences Center, New Orleans, LA 70112 USA; 3 Stanley S. Scott Cancer Center, Louisiana State University Health Sciences Center, New Orleans, LA 70112 USA; 4 Tulane University School of Medicine, New Orleans, LA 70112 USA; 5 Center for Experimental Infectious Disease Research, Louisiana State University, Baton Rouge, LA 70803 USA; 6 Present Address: Universidad Popular Autonoma del Estado de Puebla, UPAEP, Puebla, Mexico

**Keywords:** micro RNA, Pneumovirus, HMPV, RSV, Dendritic cells, DC

## Abstract

**Objective:**

Human metapneumovirus (HMPV) and respiratory syncytial virus (RSV) are responsible for respiratory diseases, mostly in children. Despite the clinical and epidemiological similarities between these two pneumoviruses, they elicit different immune responses. This work aims to further our understanding of the differential immune response induced by these respiratory viruses by determining the changes of small non-coding RNAs (miRNAs), which regulate gene expression and are involved in numerous cellular processes including the immune system.

**Results:**

In the present study, we analyzed the expression of miRNA transcripts of human dendritic cells infected with RSV or HMPV by high throughput sequencing using Illumina sequencing technology. Further validation of miRNA expression by quantitative polymerase chain reaction indicated that HMPV infection up-regulated the expression of 2 miRNAs (hsa-miR-182-5p and hsa-miR-4634), while RSV infection induced significant expression of 3 miRNAs (hsa-miR-4448, hsa-miR-30a-5p and hsa-miR-4634). The predominant miRNA induced by both viruses was hsa-miR-4634.

## Introduction

Human metapneumovirus (HMPV) and respiratory syncytial virus (RSV) are important pathogens causing upper and lower respiratory tract infections in children globally. Several epidemiological studies indicate that RSV is one of the major responsible viral pathogen for respiratory infections followed by HMPV [1–6]. The infections produced by these two pneumoviruses [7] are often clinically indistinguishable [6, 8, 9]. However, several studies indicate that RSV and HMPV differentially activate immune cells, such as dendritic cells (DCs) in vitro [10, 11] and in vivo [12]. In that regard, we and others have reported that RSV and HMPV induce differential activation of DCs, including changes in the expression of interferon [10] and costimulatory molecules in DCs [10, 12], which resulted in distinct activation of T cell responses by these two viruses [10–12].

To further understand the differential activation of DCs by RSV and HMPV, we investigated herein whether these viruses induce distinct profiles of microRNAs (miRNAs), a kind of small non-coding RNA molecules of 19-24 nucleotide in length [13] that regulate the gene expression at the transcriptional or post-transcriptional level [14]. In fact, miRNAs are known as critical regulators of several DC functions, including maturation, antigen presentation, and cytokine production [15]. In this work we analyzed the miRNA profile induced by RSV and HMPV infection in human monocyte-derived dendritic cells (moDCs). We found that RSV induced significant overexpression of hsa-miR4448, hsa-miR-30a-5p and hsa-miR-4634, while HMPV infection induced overexpression of hsa-miR-182-5p and hsa-miR-4634, providing evidence that RSV induces a profile of miRNA distinct from that of HMPV in human moDCs. Relevance of the validated miRNA is discussed.

## Main text

### Methods

#### Virus stocks

HMPV (strain CAN97-83) and RSV (strain A2) were propagated and titrated as we previously described [10, 16]. Briefly, HMPV was propagated in LLC-MK2 cells (American Type Culture Collection, CCL7) in MEM (without serum) containing 1 μg trypsin/ml (Worthington) and purified by polyethylene glycol precipitation, followed by centrifugation on a 60% sucrose cushion. Virus titer was determined by a combined method of methylcellulose plaque assay and cell-based immunoassay in LLC-MK2 cells.

RSV was grown in HEp-2 cells (ATCC CCL-23) and purified by polyethylene glycol precipitation, followed by centrifugation on 35–65% discontinuous sucrose gradients as described elsewhere [17, 18]. The virus titer was determined by a methylcellulose plaque assay in HEp-2 cells [19].

#### Establishment and infection of human monocyte-derived dendritic cells

Buffy coats used as a source of human peripheral blood mononuclear cells (PBMCs) were provided from healthy donors by the Our Lady of the Lake Blood Donor Center. The investigators did not have access to any of the donors’ identifiable information neither interacted with the donors. Blood samples were not collected by the blood center specifically for this study. The use of these samples was not considered human subjects research under the US Department of Health and Human Services (HHS) human subject regulations (45 CFR Part 46) [20]. Monocyte-derived dendritic cells (moDCs) were generated from human PBMCs, as previously described [10, 21, 22]. Briefly, adherent cells were selected from PBMCs and cultured for 7 days in RPMI 1640 medium in the presence of granulocyte–macrophage colony-stimulating factor (GM-CSF; 100 ng/ml) and interleukin-4 (IL-4; 20 ng/ml). For viral infection of the cells, moDCs from each donor were used as an independent experiment where all 3 conditions (uninfected, RSV- and HMPV-infected) were included. 1 × 10^6^ moDCs were infected with RSV or HMPV at a multiplicity of infection (MOI) of 3, or were left uninfected (control), for 24 h before RNA extraction.

#### RNA extraction

RNA from each experiment (uninfected, RSV- and HMPV-infected moDCs) was isolated using the RNeasy-plus kit (Qiagen). For miRNA sequencing, RNA from 4 independent experiments were pooled and used for the analysis. For validation of miRNA expression, RNA from 13 independent healthy donors was extracted and further tested individually by qRT-PCR.

#### Sample miRNA library preparation and deep sequencing

Small RNA libraries were generated from 1μg of total RNA using the TruSeq small RNA sample preparation kit (Illumina, San Diego, CA) and according to manufacturer’s instructions. Briefly, specifically modified 3′ and 5′ adapters that target microRNAs were first sequentially ligated to the RNA, followed by reverse transcription and PCR amplification with indexed primers. PCR products were then pooled together and run in a 6% TBE gel for gel purification. The small RNA fraction of approximately 140–160 bp in size was excised from the gel and eluted overnight. Libraries were validated on Agilent’s 2100 bioanalyzer (Agilent Technologies) using a High Sensitivity DNA Chip and assessed for the presence of the peak corresponding to ligated and tagged miRNA (~ 147 nt). The sequencing was performed in the Illumina’s Genome Analyzer II (Illumina Inc) at the Translational Genomics Core, Stanley S. Scott Cancer Center, LSUHSC-New Orleans.

#### microRNA analysis

Data processing and alignment was done at the Cancer Crusaders Next Generation Sequence Analysis Core. For data analysis, FASTQ files were generated using CASAVA v1.8.1. Twenty-nine base pair (29 bp) single-end reads were assessed for quality using software (version 0.9.6). Reads were then aligned to mature human microRNAs, release 21, from miRBase (http://www.mirbase.org) using miRNAkey v1.2 [23]. Prior to alignment with miRNAkey, Illumina miRNA adapter sequence TGGAATTCTCGGGTGCCAAGG was trimmed from reads. Similarly, 5 nucleotides from the 5′ end were also trimmed. EBSeq (http://bioconductor.org/packages/release/bioc/html/EBSeq.html) was used to perform differential expression testing to identify differentially expressed microRNAs at a false discovery rate (FDR) of 5% or less. EBSeq analysis allowed to determine the probability of equally or differentially expressed genes (PPEE and PPDE, respectively) between two conditions (infected over uninfected cells). The cut-off point selected was PPDE of 0.95 which, in our analysis, corresponds to a posterior fold change (PostFC) of 20. Raw counts of the differentially expressed genes (among all conditions) were transformed to obtain the log2 fraction and used to generate non-supervised, hierarchical heatmaps in the software Pretty Heatmaps (pheatmaps) v1.0.8 contained in the R-Studio package v1.1.383. The heatmap application groups the samples based on the level of the expression of the gene input.

#### Quantitative real-time reverse transcription-PCR (qRT-PCR) of miRNA

qRT-PCR was performed with the MystiCq™ MicroRNA™ Quantitation System (Sigma-Aldrich Merck). Briefly, cDNA was synthesized with MystiCq™ microRNA cDNA Synthesis Mix Kit according to manufacturer’s instructions. RT-qPCR was performed in triplicate using 10 nM of each MystiCq microRNA qPCR Assay Primer and 10 nM of MystiCq Universal PCR Primer plus MystiCq microRNA SYBR Green qPCR Ready Mix. qRT-PCRs were run on the 7900HT fast real-time PCR system following the manufacturer’s suggested cycling parameters (Sigma-Aldrich Merck). The comparative cycle threshold (ΔΔCT) [24] was used to quantitate the expression of target miRNAs, which were normalized to the endogenous reference expression levels of transcripts from uninfected cells (RNU6-1) [25].

#### Statistical analysis

Statistical analyses were performed with the InStat 3.05 biostatistics package (GraphPad, San Diego, CA) using one-way analysis of variance (ANOVA) followed by Dunet post-test to analyze differences between HMPV- and RSV-infected cells against uninfected cells. Results are expressed as mean ± standard error of the mean. *P *≤ 0.05 was considered statistically significant.

### Results

#### Profiling of miRNA expression in RSV- and HMPV-infected moDCs

**Fig. 1:**
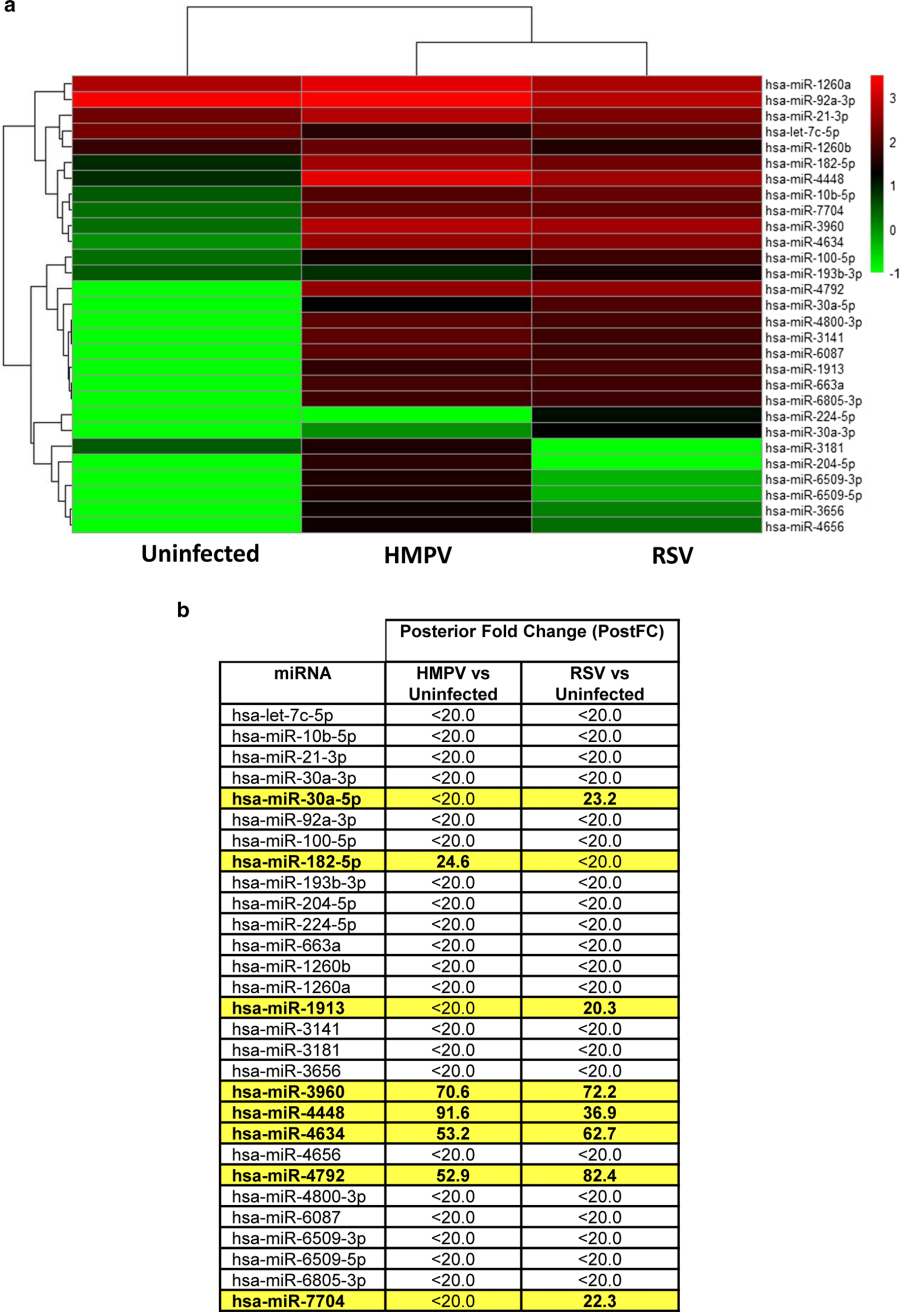
Heatmap and hierarchical clustering of the miRNAs with significant expression variance. **a** Heatmap and cluster dendrogram of differentially expressed 29 miRNAs from moDC uninfected, or infected with RSV or HMPV. Rows represent each miRNA as a log2-transformed expression of raw counts of the differentially expressed miRNAs from different conditions indicated on the bottom of each column. The color scale shown on the right-hand side illustrates the relative expression level of the indicated miRNA across all samples: red and green indicate high and low expression levels, respectively. **b** Posterior fold change determined for each miRNA in HMPV or in RSV infection over uninfected cells

In order to assess the differential activation of DCs infected with RSV or HMPV infection, microRNA expression was investigated by high-throughput sequencing (deep sequencing) analysis using Illumina platform. We conducted the clustering for each of the three culture conditions by hierarchical cluster and observed that the miRNA transcriptome of these three groups were significantly different. The hierarchical organization of the miRNAs detected in uninfected, HMPV- and RSV-infected moDCs is depicted in the heatmap shown in Fig. 1a.

#### miRNA differentially expressed by RSV and HMPV infection

In order to screen out some differentially expressed miRNA, sequences that occurred 20 or more times in infected cells were further validated. Therefore, a posterior fold change (PostFC) value below 20 was considered as a cut off for miRNA discrimination when comparing the uninfected moDCs with those infected with RSV or HMPV. Based on this criteria, we found that 8 miRNAs (hsa-30a-5p, hsa-miR-182-5p, hsa-miR-1913, hsa-miR-3960, hsa-miR-4448, hsa-miR-4634, hsa-miR-4792, hsa-miR-7704) were expressed in the infected cells when compared with the uninfected ones. On the other hand, 21 miRNAs were excluded from further analysis based on their low PostFC value (Fig. 1b).

#### Validation of miRNA expression by qRT-PCR

**Fig. 2:**
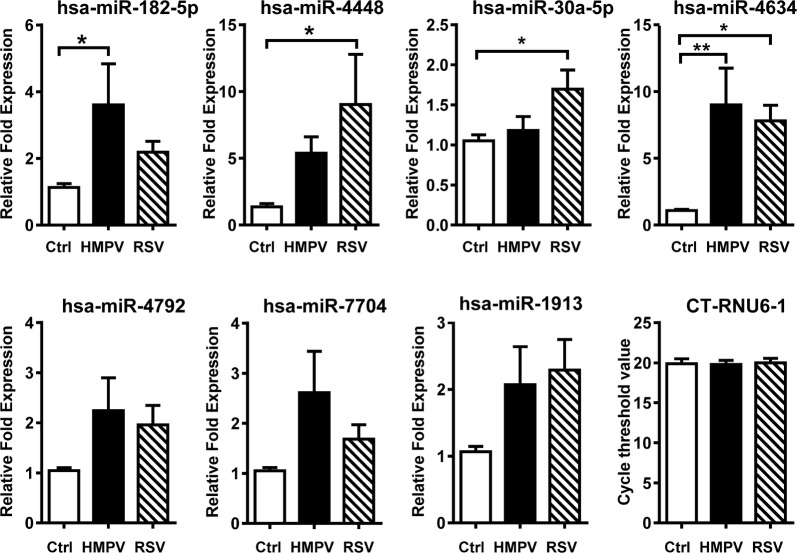
Profile of miRNAs upregulated in MoDCs infected by HMPV and RSV. Cells were infected at MOI of 3 for 24 h; miRNAs expression was assayed by qRT-PCR. Graph bars represent mean of 13 independent donors ± SEM. Statistical significant differences between infected and uninfected cells were calculated by one-way analysis of variance (ANOVA) followed by Dunet post-test and are indicated *P < 0.05; **P < 0.01

Based on their expression levels and fold difference in expression (PostFC), the aforementioned 8 miRNA were selected for validation by qRT-PCR. A total of 13 individual cultures of moDCs were included for the validation experiments. moDCs were infected with HMPV or RSV at an MOI of 3 for 24 h, followed by RNA extraction and evaluation of miRNA expression by RT-qPCR assays, as described in methods. Our data show that all the miRNAs examined, with the exception of hsa-miR-3960, were expressed in moDCs. Furthermore, when comparing uninfected moDCs vs HMPV-infected cells, the expression of hsa-miR182-5p and hsa-miR-4634 showed statistical difference in a manner consistent with the data from deep sequencing analysis. In the case of RSV, RT-qPCR assays validated the expression of hsa-miR-4448, hsa-miR-30a-5p, and hsa-miR-4634 in the infected cells. Expression of RNU6-1, as internal control, was detected in similar levels in moDCs uninfected or infected with either virus (Fig. 2).

### Discussion

Micro RNAs (miRNAs) represent a large family of highly conserved small noncoding single-stranded RNAs that are known to play a key post-transcriptional role in gene expression [13]. Indeed, these molecules are known to control about 60% of the human genome [26]. Dendritic cells are known to differentially respond to RSV and HMPV infection in vitro and in vivo [10, 12]. In the present work, we further demonstrate that RSV and HMPV infection differentially induce the expression of miRNA profile in human dendritic cells.

Deep sequencing and validation experiments indicate that RSV induced significant expression of hsa-miR-4448 in moDCs. According to functional analysis reported in the miRTarBase, CCDC88A is one of the target genes for hsa-miR-4448 [27]. CCDC88A gene codes for GIV (also known as Girdin), a protein that is associated with the regulation of the Akt/mTOR signaling pathway [28]. RSV is known to decrease the expression of p53 through a mechanism involving the activation of the Akt pathway, which results in prolonged cell survival and decreased apoptosis [29]. However, whether miR-4448 plays a role in cell survival after RSV infection, remains to be investigated. RSV infection also up-regulated the expression of hsa-miR-30a-5p. Based on the miRTarBase, KEGG pathway and gene ontology enrichments indicate that hsa-miR-30a-5p targets at least 3 genes: NOTCH1, BECN1 and NT5E [27]. Notch-1 is known to play a key role in DC differentiation [30, 31], while beclin-1 participates in autophagy, which has been shown to be necessary for inducing innate and adaptive immune responses to RSV infection [32, 33]. The upregulation of beclin-1 by RSV infection has been reported in epithelial cells [34], and its downregulation leads to suppression of RSV replication [35]. However, the role of NT5E has not been described in RSV infection, but in other respiratory viruses such as influenza, it has been reported that NT5E contributes to the induction of the neutrophil response in the airways of infected mice [36].

Furthermore, we observed that HMPV induced the expression of hsa-miR-182-5p. Several target genes have been reported for hsa-miR-182-5p: CDKN1A, FOXO1, MITF, CYLD, BCL2, CCND2, PDCD4, and RECK [27]. However, the role of expression of those genes in HMPV infection has not yet been defined. HMPV, as well as RSV induced the expression of hsa-miR-4634. Although no validated target genes have been reported for hsa-miR-4634 [27], at least four predicted targets genes for hsa-miR-4634, have been reported: bromodomain and PHD finger containing-1 (BRPF1), cyclin-dependent kinase inhibitor 2A (CDKN2A), myelin basic protein (MBP) and toll-like receptor 8 (TLR8) [37]. Despite that very limited information of hsa-miR-4634 is currently found in the literature, the expression of hsa-miR-4634 has been linked to breast cancer [38].

There are limited reports regarding the expression and/or function of miRNAs during RSV and HMPV infection. In that regard, viral replication in A549 cells has been reported to be regulated by the expression of let-7f [39, 40] in both RSV or HMPV-infected cells, while miR-24 [40] and miR-29a [41] only regulated RSV-infected cells. In addition, miR-221 affects RSV replication in primary human bronchial epithelial cells [42]. Comparative experiments of RSV infection in moDCs and epithelial cells demonstrated a cell-type specific miRNA expression. Expression of hsa-let-7c, hsa-let-7i, and hsa-miR-30b were upregulated in epithelial cells, while hsa-let-7b was overexpressed in moDCs. Although in the present work we did not observe changes in the expression of let-7b, when comparing uninfected and RSV-infected moDC, changes in hsa-miR-4448, hsa-miR-30a-5p and hsa-miR-4634 were demonstrated. We therefore speculate that the differences in the experimental strategy and miRNA analysis contributed to the observed discrepancies between the reported work [43] and the current study.

In conclusion, this work represents the first evidence of the differential expression of miRNA profiles between RSV and HMPV infection in human dendritic cells. The functional analysis of the miRNAs, their contribution to viral infection, replication and their role in shaping the immune responses warrant further investigations. These studies are critical to further our understanding of the host–pathogen interaction between immune cells and RSV and HMPV.

## Limitations

In the present work, the miRNA analysis was limited to their expression after RSV or HMPV infection. Further research focused on the functional analysis is warranted.

## Abbreviations

CCDC88A: the coiled-coil domain containing 88a
GIV: Gα-interacting, vesicle associated protein
CDKN1A: cyclin dependent kinase inhibitor 1A
FOXO1: forkhead box protein 1
MITF: melanogenesis associated transcription factor
CYLD: CYLD lysine 63 deubiquitinase
BCL2: B cell lymphoma 2
CCND2: cyclin D2
PDCD4: programmed cell death 4
RECK: reversion inducing cysteine rich protein with kazal motifs
NOTCH1: neurogenic locus notch homolog-1
BECN1: beclin-1
NT5E: and ecto-5′-nucleotidase
